# Understanding the Nucleophilic Character and Stability of the Carbanions and Alkoxides of 1-(9-Anthryl)ethanol and Derivatives 

**DOI:** 10.3390/molecules180910254

**Published:** 2013-08-22

**Authors:** Ramsés E. Ramírez, Cirilo García-Martínez, Francisco Méndez

**Affiliations:** 1 Departamento de Fisicomatemáticas, Facultad de Ciencias Químicas, Av. San Claudio y 14 Sur, Col. San Manuel, Benemérita Universidad Autónoma de Puebla, C.P. 72570, Puebla, Pue., Mexico; E-Mail: ramses.ramirez@correo.buap.mx; 2 Departamento de Química, División de Ciencias Básicas e Ingeniería, Universidad Autónoma Metropolitana-Iztapalapa, A.P. 55-534, México, D. F., 09340 Mexico; 3 Área de Química, Departamento de Ciencias Básicas, Universidad Autónoma Metropolitana-Azcapotzalco, San Pablo #180, Col. Reynosa, Mexico, D. F., 02200 México; E-Mail: gmc@correo.azc.uam.mx

**Keywords:** absolute gas phase acidity, alkoxide, carbanion, Fukui function, nucleophile

## Abstract

The nucleophilic character and stability of the carbanions *vs*. alkoxides derived from 2,2,2-trifluoro-1-(9-anthryl)ethanol and 1-(9-anthryl)ethanol containing X electron-releasing and X electron-acceptor substituents attached to C-10, have been studied at the B3LYP/6-31+G(d,p) level of theory. Results analyzed in terms of the absolute gas-phase acidity, Fukui function, the local hard and soft acids and bases principle, and the molecular electrostatic potential, show that the central ring of the 9-anthryl group confers an ambident nucleophilic character and stabilizes the conjugated carbanion by electron-acceptor delocalization.

## 1. Introduction

**Figure 1:**
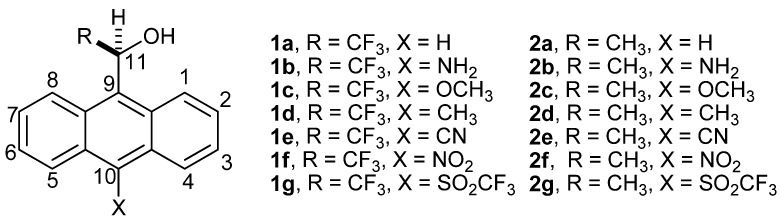
1-(9-anthryl)ethanol and derivatives.

Pure enantiomers of 2,2,2-trifluoro-1-(9-anthryl)ethanol (**1a**, Figure 1), are mainly used as chiral solvating agents [1,2] and chiral selectors [1,2,3,4] due to their particular hydroxyl (OH) and methine (CH) acidity [5,6,7,8]. The calculated gas phase acidities for **1a**, 1-(9-anthryl)ethanol (**2a**) and 2,2,2-trifluoroethanol have shown that the influence of 9-anthryl ring is more significant than the trifluoromethyl group in modifying the CH rather than the OH acidity of **1a**; the trifluoromethyl group increases 6.0 kcal mol^−1^ the OH acidity more than the CH acidity, while the 9-anthryl group increases 17.0 kcal mol^−1^ the CH acidity more than the OH acidity [6].

**Scheme 1:**
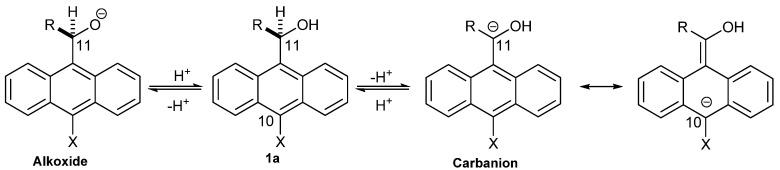
The resonance effects on the OH and CH ionization and the ambident nucleophilic character for the carbanion.

These results imply that the anthryl ring stabilizes more efficiently the negative charge in the carbanion than the alkoxide (Scheme 1). The resonance effects on the alkoxide are absent; while the resonance effects on the carbanion are present and the negative charge ought to be delocalized mainly on the C-11 and C-10 carbon atoms. Therefore, an ambident nucleophilic character should be observed for the carbanion, and there would be an electrophilic attack on the C-11 and C-10 carbon atoms.

The proposal is consistent with the ambident nucleophilic character experimentally observed for the 9-anthrylmethyl carbanion and related intermediates [9]. Also, it has been reported that the electrophilic attack on (9-anthryl)phenylmethyl and related anions gave the mixture of the two expected products (electrophilic addition on the C-11 and C-10 carbon atoms), whose composition depended of the substituent attached to the phenyl ring of the substrate [10]. However, experimental studies concerning the acidity of OH and CH of **1a** and **2a**, the ambident nucleophilic character and the origin of the stability for their carbanions *vs*. alkoxides have not been carried out so far. Therefore, we take **1a**, **2a**, and their derivatives containing X electron-releasing **1b**–**1d** and **2b**–**2d**, and X electron-acceptor **1e**–**1g** and **2e**–**2g** substituent attached to C-10 (Figure 1) as a set of alcohols. The Fukui function, a density functional theory (DFT) descriptor [11,12], the local hard and soft acids and bases principle (HSAB) [13,14], and the molecular electrostatic potential (MEP) [15,16] were used to demonstrate, in terms of the electron density, that the resonance effects in the carbanions are responsible for the ambident nucleophilic character and stability with respect to the alkoxides, due to the delocalization of the electron density on the C-11 and C-10 carbon atoms.

## 2. Results and Discussion

Table 1 shows the Δ_acid_*G°*(CH) and Δ_acid_*G°*(OH) values obtained for the ionization reactions shown in Scheme 1 for **1b**–**1g** and **2b**–**2g**. The calculated acidity values are consistent with the substituent electronic effect, being the alcohol with X = NH_2_ (compounds **1b**, **2b**) the least acidic and with X = SO_2_CF_3_ (compounds **1g**, **2g**) the most acidic; they correspond to the two extremes for which Δ_acid_*G°*(CH) and Δ_acid_*G°*(OH) values decrease over 40 and 18 kcal/mol, respectively. The CH acidity is higher than the OH acidity for **1e**–**1g** and **2b**–**2g** (positive δΔ_acid_*G°* values), which implies that the carbanions **1e**–**1g** and **2b**–**2g** are more stable than the alkoxides by 3.1–12.4 and 3.8–26.1 kcal mol^−1^ respectively.

**Table 1:** Absolute gas phase acidities for alcohols **1b**–**2g**. Values are reported in kcal mol^−1^. δΔ_acid_*G°* = Δ_acid_*G°*(OH) − Δ_acid_*G°*(CH).

Alcohol	Δ_acid_*G°*(CH)	Δ_acid_*G°*(OH)	δΔ_acid_*G°*
**1b (2b)**	349.8 (352.3)	342.1 (356.2)	−7.7 (3.9)
**1c (2c)**	346.3 (347.8)	339.3 (353.4)	−7.0 (5.6)
**1d (2d)**	345.1 (348.0)	339.9 (353.3)	−5.2 (5.3)
**1e (2e)**	327.2 (328.5)	330.3 (344.1)	3.1 (15.6)
**1f** **(2f)**	319.9 (318.6)	330.5 (343.7)	10.6 (25.1)
**1g (2g)**	313.1 (311.9)	325.6 (338.0)	12.5 (26.1)

Negative δΔ_acid_*G°* values for **1b**–**1d** indicate that the electron-releasing substituent is not efficient enough to delocalize the negative charge into the aromatic ring; the carbanions of **1b**–**1d** are less stable than the alkoxides by 7.7–5.2 kcal mol^−1^ respectively. Therefore, the stability of the carbanions of **1** and **2** increases as the electron-acceptor capacity of the substituent increases [17]. The correlation between the electronic effect of X and the ionization reactions of Scheme 1, is evident from the good linear regression analysis obtained for Δ_acid_*G°*(CH) or Δ_acid_*G°*(OH) and the Hammett substituent constants in the gas σ_p_^−^ (g) or aqueous σ_p_^−^ (aq) phase (see Table 2) [18]. The slope values are quite large and negative for Δ_acid_*G°*(CH) as would be expected for the generation of a large amount of negative charge upon the ring. On the other hand the lower slope values for Δ_acid_*G°*(OH) indicates localized negative charge on oxygen atom, but not transmittable to the ring [19].

**Scheme 2:**
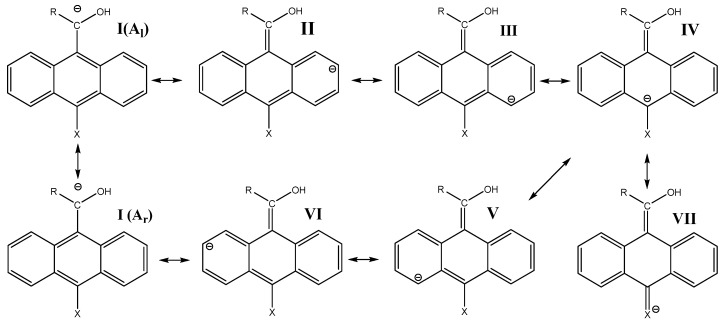
The Kekulé resonance structures for the carbanion.

While, the resonance effects on the CH ionization are present, the delocalization of the negative charge of the carbanions into the ring can be illustrated in terms of resonance theory [20]. The Kekulé resonance structures **I**–**VII** on Scheme 2 show that the negative charge is localized on the C-2 (**II**), C-4 (**III**), C-10 (**IV**), C-5 (**V**), C-7 (**VI**), C-11(**I**) carbon atoms, and on the substituent X (**VII**). The classical Kekulé structures **A**_l_ and **A**_r_ are structures in which double bond alternation extends over the whole periphery of the molecule [21].

**Table 2:** Linear correlation equation between Δ_acid_*G°*(CH) or Δ_acid_*G°*(OH) and σ_p_^−^_(g)_, σ_p_^−^_(aq)_ or 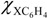 for **1b**–**1g**. The linear correlation equation for **2b**–**2g** is given in parenthesis. Δ_acid_*G*° = b + mY(values in kcal mol^−1^).

Y	Δ_acid_ *G*°	b	M	R^2^
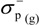	CH	344.9	−23.0	0.99
		(347.1)	(−25.2)	(0.98)
	OH	339.2	−9.7	0.96
		(353.3)	(−1.0)	(0.97)
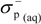	CH	345.6	−20.1	0.97
		(343.6)	(−18.2)	(0.98)
	OH	338.9	−7.7	0.95
		(352.7)	(−8.3)	(0.95)
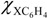	CH	472.5	−24.7	0.98
		(488.0)	(−27.3)	(0.97)
	OH	393.3	−10.4	0.98
		(409.2)	(−10.8)	(0.99)

**Figure 2:**
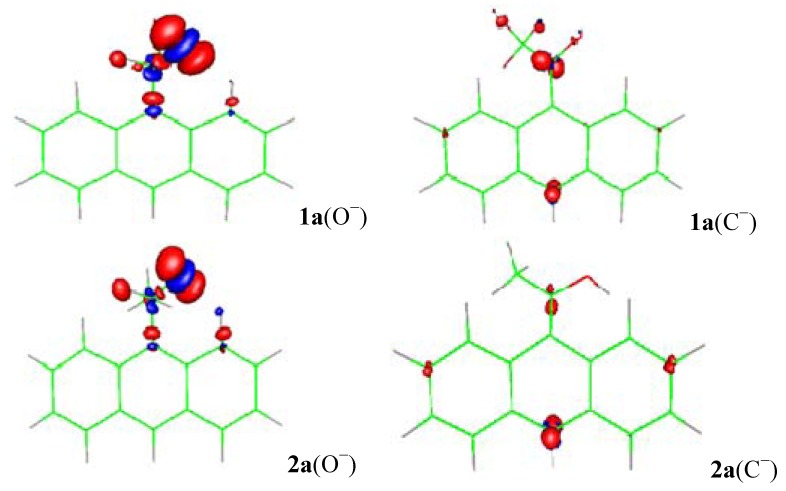
The surface plot of the electrophilic Fukui function for the alkoxides (O^−^) and carbanions (C^−^) of **1a** and **2a**.

**Figure 3:**
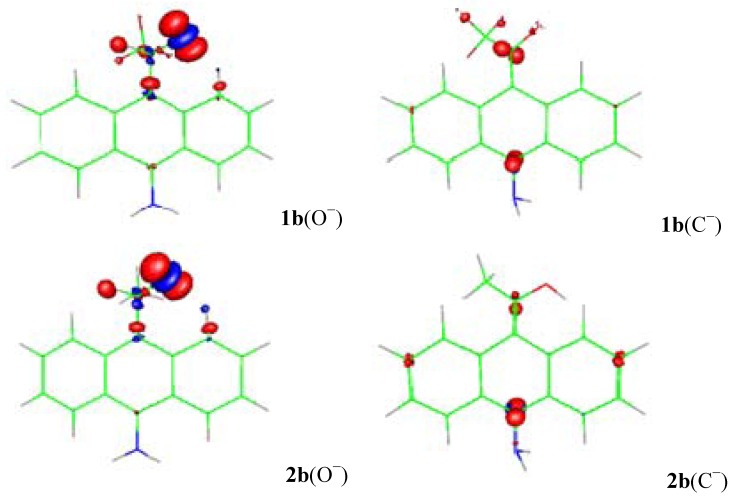
The surface plot of the electrophilic Fukui function for the alkoxides (O^−^) and carbanions (C^−^) of **1b** and **2b**.

**Figure 4:**
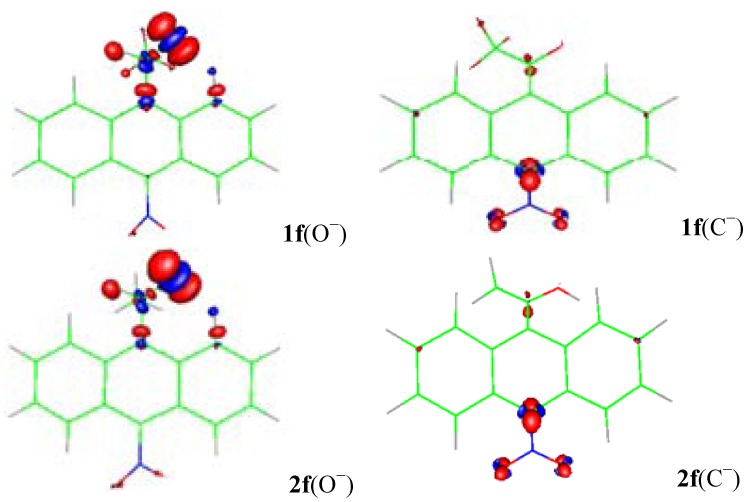
The surface plot of the electrophilic Fukui function for the alkoxides (O^−^) and carbanions (C^−^) of **1f** and **2f**.

We have shown previously that the resonance hybrid structure can be described in terms of the Fukui function [22]. The Fukui function *f*(**r**) represents the change of the electronic density *ρ*(**r**) in a given point with respect to the change in the number of electrons N, *f*(**r**) = (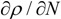), and the stability of the ion can be achieved from the link between *f*(**r**) and the energy E of the system *f*(**r**) = 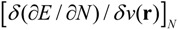 [11,12], where *v*(**r**) is the external potential associated to the nucleus. The amount of charge in the carbanions and alkoxides was examined specifically from the Fukui function for electrophilic attack *f*^−^(**r**) (Figure 2, Figure 3 and Figure 4) and the local HSAB principle [13,14]. Figure 2 shows that the largest values of *f*^−^(**r**) for **1a** and **2a** are located on the O and the C-11/C-10 atoms for the alkoxides and carbanions respectively. They are associated with softer nucleophilic regions (*s*^−^(**r**) = S*f*^−^(**r**)), by giving up electronic charge and are especially reactive toward soft electrophiles [13]. Therefore, charge localization occurs on the strongly electronegative oxygen atom for the alkoxides; whereas, the charge is located on the C-11/C-10 carbon atoms indicating an ambident soft nucleophilic character for the carbanions and the distribution of the anionic charge into the ring is represented in terms of the resonance structures **I** and **IV**.

When the NH_2_ electron-releasing substituent is included (Figure 3), the behavior of the *f*^−^(**r**) for **1b** and **2b** is almost similar to that of **1a** and **2a**; the largest values of *f*^−^(**r**) are located on the O and the C-11/C-10 atoms. The NH_2_ substituent has no contribution to the charge dispersal. For the alkoxide charge localization occurs on the strongly electronegative oxygen atom; whereas, for the carbanion the charge dispersion involved the C-11/C-10 carbon atoms indicating an ambident soft nucleophilic character that is well represented by resonance structures **I** and **IV** (Scheme 2). The generation of a small amount of negative charge upon the ring makes the carbanion of **1b** 7.7 kcal mol^−1^ less stable than the corresponding alkoxide. The carbanion of **2b** delocalizes the negative charge into the aromatic ring more efficiently than that of **1b** due the *f*^−^(**r**) trends for **1b** and **2b** are C-11 > C-10 and C-11 < C-10 respectively; the carbanion of **2b** is 3.9 kcal mol^−1^ more stable than the corresponding alkoxide (See Table 1). The results are similar for X = OCH_3_ and CH_3_ (See Figures in the Electronic Supplementary Information).

When the NO_2_ substituent is included (Figure 4), *f*^−^(**r**) for the alkoxides of **1f** and **2f** shows localized negative charge on oxygen atom, but not transmittable to the ring. For the carbanions of **1f** and **2f**, *f*^−^(**r**) indicates generation of a large amount of negative charge localized on C-10 and on the NO_2_ substituent (with most of the negative charge residing on the two oxygen atoms) [23,24,25], making the carbanions of **1f** and **2f** 10.6 and 25.1 kcal mol^−1^ more stable than their alkoxides respectively (see Table 1). Therefore, the distribution of the anionic charge into the ring is represented in terms of the resonance structures **IV** and **VII** (Scheme 2). The results are similar for X = CN and SO_2_CF_3_ (see figures in the Supplementary Information). 

As we can observe, the Fukui function for electrophilic attack shows that delocalization of the anionic charge mainly occurs in the central ring of the 9-anthryl group, making the central ring more reactive than the edge rings for soft-soft interactions. This important result can be supported by the electronegativity of the central ring effect of the 9-anthryl group on the Δ_acid_*G°*(CH) or Δ_acid_*G°*(OH). 

The electronegativity of the central ring can be approximated by the XC_6_H_4_ fragment electronegativity 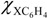 calculated previously for a set of *p*-substituted phenols XC_6_H_4_OH [7]. From Table 2 we can observe the negative slope values; the acidity increases (Δ_acid_*G°* decreases) when 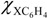
increases, validating the proposal that the central ring exhibits more contribution to Δ_acid_*G°* than the edge rings and it behaves as a rather separated benzenoid ring or localized entity. This is a nice coincidence with the ambident nucleophilic character experimentally observed for the 9-anthrylmethyl carbanion and related intermediates [26], and the calculated aromaticity of the central ring of anthracene [27,28,29].

**Figure 5:**
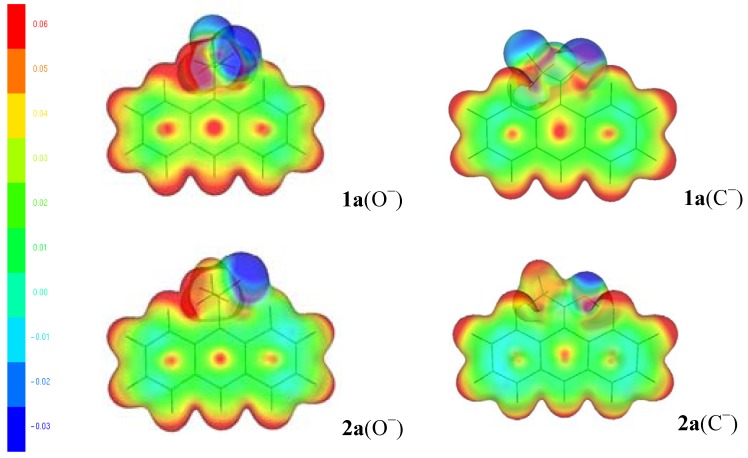
MEP mapped onto an isosurface of the total electron density for the alkoxides (O^−^) and carbanions (C^−^) of **1a** and **2a**.

**Figure 6:**
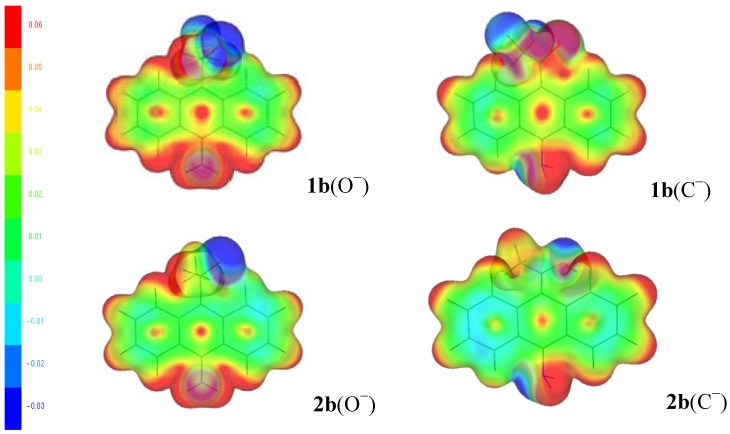
MEP mapped onto an isosurface of the total electron density for the alkoxides (O^−^) and carbanions (C^−^) of **1b** and **2b**.

**Figure 7:**
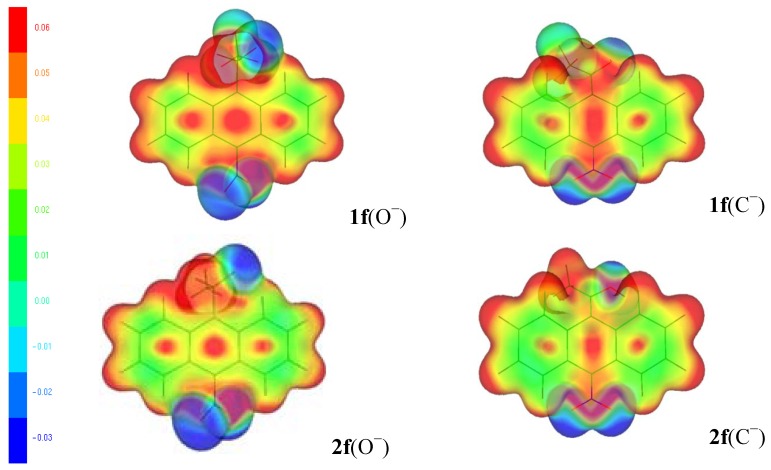
MEP mapped onto an isosurface of the total electron density for the alkoxides (O^−^) and carbanions (C^−^) of **1f** and **2f**.

Figure 5, Figure 6 and Figure 7 show the molecular electrostatic potential (MEP) [15,16] mapped onto an isosurface of the total electron density for the alkoxides and carbanions of **1a**–**2a**, and **1f**–**2f** respectively. The MEP can be appropriate for analyzing hard-hard interactions because the proton (a hard electrophile) prefers a site with maximum net charge [13,30]. In Figure 5, we observe the MEP for the alkoxides and carbanions of **1a** and **2a**, in these anions, the blue area, towards the oxygen (**1a** and **2a**) and fluorine atoms (**1a**), indicates higher negative charge; the red color, located near the hydrogen atoms and methyl group indicate more positive charge and lower electron density. 

We notice an uneven distribution of electrons with positive charge in the 9-anthryl ring, the central ring exhibits more positive charge than the edge rings. That is, in the alkoxides and carbanions of **1a** and **2a,** the hydrogen atoms are more positive (red color) than the carbon atoms of the 9-anthryl ring and the C-11 carbon atom, and the oxygen and fluorine atoms are more negative (blue colors). When the NH_2_ electron-releasing substituent is included (Figure 6), the behavior of the MEP for the **2b**, the hydrogen atoms are more positive (red color) than the 9-anthryl ring and the C-11 carbon atom. The oxygen, nitrogen and fluorine atoms are more negative (blue colors). The results are similar for X = OCH_3_ and CH_3_ (see figures in the Supplementary Information).

In Figure 7, we observe the MEP for the alkoxides and carbanions of **1f** and **2f**, in these anions, the blue negative area is located towards the oxygen, nitrogen (compounds **1f** and **2f**) and fluorine atoms (**1f**); the red color is located near the hydrogen atoms, methyl group, C-11 carbon atom and the 9-anthryl ring. The uneven distribution of electrons with positive charge in the 9-anthryl ring is observed, the central ring exhibits more positive charge than the edge rings, indicating lower electron density than in the anions of **1a**–**2a** and **1b**–**2b**. The results are similar for X = CN and SO_2_CF_3_ (see figures in the Supplementary Information). Therefore, the proton (a hard electrophile) prefers the oxygen (alkoxides **1** and **2**) and fluorine atoms (alkoxide **1**) with maximum MEP (the blue area with higher negative charge).

## 3. Theoretical Methodology

The ground state structures and energies of alcohols **1a**–**2g** were calculated at the B3LYP/6-31+G(d,p) level of theory using GAUSSIAN03 [31]. The thermodynamic stability of the carbanions and alkoxides of **1a**–**2g** was obtained from the calculated CH and OH absolute gas-phase acidities [32]. Gas phase studies are required to separate intrinsic molecular properties from interfering solvation effects [33]. The absolute gas-phase acidity Δ_acid_*G°* is given by Δ_acid_*G°* = *G°*(anion) + *G°*(H+) − *G°*(alcohol) [34]. The Gibbs free energies *G°* (anion), *G°*(alcohol) and *G°*(H+) were obtained by means of partition functions using statistical thermodynamic relationships [35]. The electrophilic Fukui function and the electrostatic potential of the molecules were visualized throughout by the gOpenmol software [36].

## 4. Conclusions

The nucleophilic character and the relative stability of the carbanions *vs*. the corresponding alkoxides of 1-(9-anthryl)ethanol and its derivatives has been studied in terms of the absolute gas phase acidity, Fukui function, the local HSAB bases principle and the molecular electrostatic potential. The central ring of the 9-anthryl group stabilizes more efficiently the conjugated carbanion than the oxygen atom of the alkoxide by electron-acceptor delocalization; as a consequence the negative charge is delocalized on the C-11 and C-10 carbon atoms that is well represented for the resonance structures **I** and **IV**. The C-11 and C-10 carbon atoms with maximum Fukui function give an ambident soft nucleophilic character to the carbanions **1** and **2**. The X substituent has an important contribution to the negative charge dispersal. Therefore, the negative charge is localized on the oxygen atom of the alkoxide, but nor transmittable to the ring; the proton (a hard electrophile) prefers the oxygen (alkoxides **1** and **2**) and fluorine atoms (alkoxide **1**) with maximum MEP (the blue area with higher negative charge) [37]. The results obtained in this work open the possibility to analyze the stability of anions as a result of the delocalization of the anionic charge in terms of the electron density. Studies related to the experimental reactivity of **1a**–**1f** are underway and will be reported in due course.

## Supplementary Materials

Supplementary materials can be accessed at: http://www.mdpi.com/1420-3049/18/9/10254/s1.
