# Supplementary material for: Understanding the Nucleophilic Character and Stability of the Carbanions and Alkoxides of 1-(9-Anthryl)ethanol and Derivatives
Source: Molecules. 2013 Aug 22;18(9):10254–65. doi: 10.3390/molecules180910254 (PMC6270014; doi:10.3390/molecules180910254)

Supplementary Materials

Computed Cartesian Coordinates and Energies of 1b−1g and 2b–2g. Calculated at the B3LYP/6−31+G(d,p) level of theory.

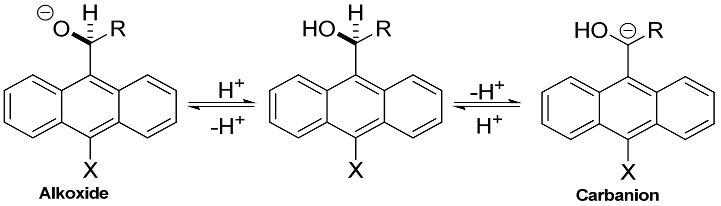

1a, R = CF_3, X = H
1b, R = CF_3, X = NH_2
1c, R = CF_3, X = OCH_3
1d, R = CF_3, X = CH_3
1e, R = CF_3, X = CN
1f, R = CF_3, X = NO_2
1g, R = CF_3, X = SO_2CF_3

2a, R = CH_3, X = H
2b, R = CH_3, X = NH_2
2c, R = CH_3, X = OCH_3
2d, R = CH_3, X = CH_3
2e, R = CH_3, X = CN
2f, R = CH_3, X = NO_2
2g, R = CH_3, X = SO_2CF_3

Carbanion 1b
E Gibbs = −1045.7339 a.u.
E scf = −1045.6894 a.u.
Enthalpy = −1045.6712 a.u.

C	0.000000	0.000000	0.000000
C	0.000000	0.000000	1.387386
C	1.237303	0.000000	2.072171
C	2.424486	0.083054	1.373127
C	2.447881	0.156852	−0.056015
C	1.194470	0.009001	−0.753924
C	1.184147	−0.086381	−2.214708
C	2.301542	0.593250	−2.889161
C	3.539630	0.767732	−2.148728
C	3.633286	0.424915	−0.777189
C	4.653934	1.327939	−2.853386
C	4.564233	1.743326	−4.168099
C	3.344863	1.627036	−4.863322
C	2.247644	1.059224	−4.222746
C	0.197255	−0.799863	−2.887084
C	−0.660106	−1.884750	−2.366155
O	−0.047499	−0.591352	−4.255118
F	−1.995885	−1.563704	−2.119493
F	−0.226647	−2.511246	−1.253500
F	−0.775380	−2.892086	−3.333177
H	−0.011077	−1.445309	−4.708435
H	−0.947455	0.004372	−0.529152
H	−0.938381	0.003296	1.935768
H	1.254901	−0.034713	3.160515
H	3.376884	0.125879	1.890377
N	4.876002	0.523389	−0.045052
H	5.591785	1.458677	−2.321399
H	5.434677	2.178961	−4.656351
H	3.255336	1.975140	−5.889371
H	1.313234	0.974635	−4.758276
H	5.207111	1.486343	0.025087
H	5.614924	−0.008222	−0.498960

Alkoxide 1b
E Gibss = −1045.7462 a.u.
E scf = −1045.7014 a.u.
Enthalpy = −1045.6836 a.u.

C	0.000000	0.000000	0.000000
C	0.000000	0.000000	1.372459
C	1.226678	0.000000	2.089738
C	2.412561	0.012507	1.399766
C	2.456668	0.030835	−0.032787
C	1.208349	0.020811	−0.779084
C	1.199567	0.038928	−2.198486
C	2.450411	0.016351	−2.869155
C	3.698136	−0.020395	−2.135264
C	3.685950	0.044771	−0.724670
C	4.928729	−0.119331	−2.864813
C	4.946953	−0.139351	−4.237376
C	3.728561	−0.046349	−4.963922
C	2.526402	0.029870	−4.305599
C	−0.143829	0.151364	−3.022617
C	−0.637915	−1.304275	−3.346867
O	−0.106267	0.912925	−4.113867
F	−1.896470	−1.293273	−3.871124
F	−0.720081	−2.131847	−2.242022
F	0.137292	−1.973568	−4.243632
H	−0.921329	0.472646	−2.288223
H	−0.949525	−0.024153	−0.516547
H	−0.943593	−0.015660	1.912222
H	1.226246	−0.025328	3.176810
H	3.337648	−0.032259	1.967194
N	4.908125	0.071433	−0.010924
H	5.867875	−0.226861	−2.329764
H	5.893672	−0.230603	−4.766050
H	3.749499	−0.034882	−6.051217
H	1.579004	0.152635	−4.825054
H	4.832403	0.522710	0.891262
H	5.657282	0.505348	−0.535128

Carbanion 1c
E Gibbs = −1104.8894 a.u.
E scf = −1104.8432 a.u.
Enthalpy = −1104.8238 a.u.

C	−1.228657	1.843777	−0.882266
C	−1.046853	3.219802	−0.865736
C	0.113554	3.753050	−0.258335
C	1.097152	2.913709	0.222920
C	0.967027	1.489807	0.138963
C	−0.289279	0.948703	−0.324158
C	−0.540186	−0.492509	−0.253113
C	0.658647	−1.345651	−0.250480

C	1.915105	−0.767065	0.186982
C	2.021199	0.614925	0.443386
C	3.044165	−1.632630	0.340975
C	2.982713	−2.972238	0.018646
C	1.784228	−3.522478	−0.483778
C	0.657370	−2.717876	−0.603049
C	−1.842400	−0.985853	−0.164148
C	−3.037785	−0.308794	0.371971
O	−2.144439	−2.311837	−0.521530
F	−3.995695	0.113345	−0.555405
F	−2.817474	0.755510	1.171444
F	−3.774133	−1.210115	1.154362
H	−2.606286	−2.732937	0.217361
H	−2.118426	1.435642	−1.349452
H	−1.788705	3.875288	−1.313820
H	0.246499	4.831739	−0.193661
H	2.000426	3.321061	0.666018
H	3.964870	−1.202218	0.720943
H	3.864197	−3.599282	0.142005
H	1.734767	−4.569678	−0.771954
H	−0.257290	−3.147265	−0.984160
O	3.240719	1.137586	0.908347
C	4.133735	1.522683	−0.134141
H	3.693568	2.300167	−0.772980
H	5.034601	1.919198	0.347926
H	4.408861	0.665478	−0.764222

Alkoxide 1c
E Gibbs = −1104.9005 a.u.
E scf = −1104.8540 a.u.
Enthalpy = −1104.8349 a.u.

C	0.000000	0.000000	0.000000
C	0.000000	0.000000	1.372901
C	1.225513	0.000000	2.097229
C	2.415934	0.019598	1.417843
C	2.451326	0.038995	−0.014009
C	1.212547	0.026440	−0.771670
C	1.227155	0.045054	−2.193803
C	2.485113	0.038777	−2.854879
C	3.722744	0.015075	−2.103178
C	3.671739	0.050726	−0.702099
C	4.974660	−0.052367	−2.796433
C	5.015712	−0.068250	−4.167207
C	3.807516	−0.001851	−4.918349
C	2.588460	0.053822	−4.289553
C	−0.108633	0.147742	−3.032916
C	−0.590268	−1.312749	−3.353551
O	−0.059825	0.905182	−4.124774
F	−1.835559	−1.310388	−3.905942
F	−0.693613	−2.128419	−2.242018

F	0.208362	−1.986956	−4.225431
H	−0.893626	0.469058	−2.306171
H	−0.948041	−0.025650	−0.519405
H	−0.944710	−0.018127	1.910635
H	1.215043	−0.022102	3.184503
H	3.360152	−0.004200	1.951762
H	5.885579	−0.115923	−2.210421
H	5.972666	−0.129700	−4.681780
H	3.852366	0.011158	−6.004910
H	1.651985	0.160181	−4.830736
O	4.861342	0.056280	0.026560
C	5.379589	1.362742	0.274376
H	4.677809	1.961862	0.870035
H	6.310034	1.229667	0.834291
H	5.590094	1.892558	−0.663578

Carbanion 1d
E Gibbs = −1029.6859 a.u.
E scf = −1029.6411 a.u.
Enthalpy = −1029.6228 a.u.

C	−0.029368	0.096719	−0.014623
C	−0.027833	0.144865	1.373117
C	1.210126	0.120886	2.050703
C	2.397579	0.121303	1.344107
C	2.431773	0.130290	−0.089738
C	1.159962	0.026865	−0.768817
C	1.131406	−0.106643	−2.225737
C	2.255296	0.538523	−2.918426
C	3.512097	0.656397	−2.200183
C	3.626789	0.298767	−0.830475
C	4.625153	1.193598	−2.926562
C	4.519640	1.625302	−4.234790
C	3.283788	1.553320	−4.907104
C	2.184225	1.015270	−4.247351
C	0.126392	−0.812850	−2.874578
C	−0.765796	−1.851902	−2.317256
O	−0.113119	−0.640877	−4.247937
F	−2.088688	−1.476211	−2.082492
F	−0.352698	−2.452041	−1.182135
F	−0.913428	−2.887190	−3.247148
H	−0.082075	−1.506910	−4.678181
H	−0.976341	0.125277	−0.544274
H	−0.962760	0.209136	1.923205
H	1.234857	0.139425	3.139176
H	3.332263	0.165539	1.893950
H	5.575836	1.307088	−2.415865
H	5.391680	2.044368	−4.734604
H	3.183972	1.915454	−5.927375
H	1.234988	0.963625	−4.761267
C	4.972915	0.295329	−0.144755

H	4.974803	−0.367527	0.727244
H	5.761701	−0.071269	−0.812247
H	5.295385	1.290695	0.211222

Alkoxide 1d
E Gibbs = −1029.6941 a.u.
E scf = −1029.6481 a.u.
Enthalpy = −1029.6300 a.u.

C	0.194976	−2.569115	0.360625
C	1.088566	−3.601177	0.229573
C	2.461926	−3.325263	−0.006427
C	2.890153	−2.025886	−0.097118
C	1.993417	−0.910176	0.035583
C	0.583584	−1.187643	0.274116
C	−0.357249	−0.131442	0.406903
C	0.107731	1.200689	0.253270
C	1.503729	1.474345	−0.023736
C	2.445675	0.421928	−0.087918
C	1.895292	2.839119	−0.243673
C	0.990555	3.868725	−0.173978
C	−0.369271	3.603734	0.143294
C	−0.793469	2.316659	0.357817
C	−1.869776	−0.408331	0.781082
C	−2.682299	−0.558339	−0.555981
O	−2.439560	0.459772	1.612043
F	−3.945253	−1.010683	−0.313949
F	−2.142628	−1.467031	−1.447156
F	−2.824735	0.597942	−1.259340
H	−1.897840	−1.458269	1.157335
H	−0.846782	−2.806850	0.523734
H	0.743312	−4.629615	0.299580
H	3.171706	−4.141467	−0.120854
H	3.939364	−1.841777	−0.295947
H	2.927085	3.064179	−0.489152
H	1.319316	4.890417	−0.355051
H	−1.075232	4.426830	0.226028
H	−1.809297	2.076412	0.660995
C	3.910779	0.742776	−0.309224
H	4.566987	−0.066156	0.014525
H	4.136768	0.944156	−1.366497
H	4.210435	1.629241	0.257871

Carbanion 1e
E Gibbs = −1082.6723 a.u.
E scf = −1089.6272 a.u.
Enthalpy = −1082.6089 a.u.

C	−0.009450	0.013816	0.008784
C	−0.006096	0.011299	1.397614
C	1.227858	0.007290	2.077794
C	2.416404	0.078180	1.373020
C	2.433951	0.138892	−0.048719

C	1.185436	0.013999	−0.739448
C	1.186626	−0.076773	−2.208830
C	2.279056	0.660676	−2.876474
C	3.524511	0.788703	−2.169576
C	3.630568	0.382240	−0.796505
C	4.627379	1.365181	−2.859800
C	4.504604	1.857237	−4.146569
C	3.264284	1.802811	−4.808791
C	2.179843	1.205204	−4.176528
C	0.264110	−0.830432	−2.900036
C	−0.634712	−1.896190	−2.368552
O	0.093291	−0.678219	−4.278011
F	−1.949219	−1.520902	−2.154262
F	−0.228128	−2.503301	−1.238173
F	−0.743575	−2.911568	−3.313238
H	0.089285	−1.552669	−4.691677
H	−0.957262	0.023630	−0.519246
H	−0.943133	0.014990	1.947602
H	1.250261	−0.016977	3.165341
H	3.362458	0.128050	1.904482
C	4.878622	0.422257	−0.138944
H	5.576468	1.443476	−2.337420
H	5.367217	2.306337	−4.634657
H	3.150721	2.215873	−5.807783
H	1.230493	1.154354	−4.690595
N	5.916723	0.449142	0.409595

Alkoxide 1e
E Gibbs = −1082.6674 a.u.
E scf = −1082.6216 a.u.
Enthalpy = −1082.6034 a.u.

C	−0.507165	−2.523324	−0.484317
C	−1.587358	−3.365893	−0.394821
C	−2.887654	−2.842715	−0.165857
C	−3.064951	−1.489500	−0.028401
C	−1.960842	−0.584969	−0.110614
C	−0.630651	−1.098508	−0.355072
C	0.487143	−0.216686	−0.449907
C	0.269429	1.182701	−0.304297
C	−1.048147	1.702681	−0.022745
C	−2.143183	0.807696	0.062469
C	−1.221412	3.107498	0.173029
C	−0.153997	3.966215	0.087476
C	1.142484	3.468808	−0.215566
C	1.347492	2.126535	−0.416133
C	1.932606	−0.760122	−0.787303
C	2.666010	−1.039089	0.574028
O	2.655619	−0.010220	−1.609938
F	3.849563	−1.676329	0.367772
F	1.956583	−1.858547	1.429709
F	2.955436	0.078707	1.292717

H	1.783520	−1.800937	−1.157603
H	0.471041	−2.950551	−0.654194
H	−1.445370	−4.438003	−0.501221
H	−3.741103	−3.512775	−0.099809
H	−4.055909	−1.083279	0.150036
C	−3.449562	1.315764	0.334079
H	−2.215350	3.485853	0.391674
H	−0.305926	5.032548	0.239359
H	1.976011	4.160523	−0.307803
H	2.307418	1.714563	−0.716598
N	−4.519727	1.728449	0.554990

Carbanion 1f
E Gibbs = −1194.9352 a.u.
E scf = −1194.8889 a.u.
Enthalpy = −1194.8700 a.u.

C	0.000000	0.000000	0.000000
C	0.000000	0.000000	1.389464
C	1.231904	0.000000	2.059878
C	2.430086	0.040926	1.357671
C	2.458843	0.086980	−0.061688
C	1.199669	0.002934	−0.732720
C	1.193977	−0.025889	−2.205425
C	2.196662	0.872061	−2.812071
C	3.468418	0.967369	−2.158996
C	3.650284	0.304180	−0.871145
C	4.450570	1.777955	−2.787777
C	4.176147	2.490018	−3.948067
C	2.913557	2.421889	−4.552751
C	1.940480	1.607815	−3.984004
C	0.373259	−0.823236	−2.948442
C	−0.492497	−1.946918	−2.456389
O	0.254383	−0.653789	−4.323020
F	−1.812603	−1.612835	−2.260001
F	−0.076478	−2.554170	−1.333616
F	−0.538595	−2.937750	−3.424715
H	0.244661	−1.523232	−4.746889
H	−0.942971	0.008481	−0.537607
H	−0.937142	0.000434	1.939737
H	1.259125	−0.009015	3.147486
H	3.369845	0.054669	1.889371
N	4.917302	−0.007392	−0.388339
H	5.427990	1.841100	−2.333187
H	4.955055	3.115169	−4.379827
H	2.695300	2.987175	−5.455327
H	0.963598	1.534378	−4.444351
O	5.049651	−0.548722	0.749847
O	5.944480	0.211097	−1.097594

Alkoxide 1f
E Gibbs = −1194.9182 a.u.
E scf = −1194.8710 a.u.
Enthalpy = −1194.8520 a.u.

C	0.000000	0.000000	0.000000
C	0.000000	0.000000	1.371408
C	1.228629	0.000000	2.081282
C	2.419058	0.004557	1.401247
C	2.457734	0.007016	−0.031229
C	1.210875	0.009399	−0.771867
C	1.202794	0.010342	−2.197688
C	2.445058	−0.013260	−2.889462
C	3.700532	−0.108476	−2.173042
C	3.659757	−0.075457	−0.763037
C	4.920367	−0.172723	−2.920255
C	4.908236	−0.138531	−4.291806
C	3.683677	−0.027262	−5.002111
C	2.494135	0.045442	−4.324218
C	−0.152303	0.116877	−3.007963
C	−0.628194	−1.344050	−3.334891
O	−0.126923	0.896138	−4.082669
F	−1.878164	−1.339812	−3.870448
F	−0.710796	−2.161344	−2.225203
F	0.163790	−2.005487	−4.220092
H	−0.922867	0.425043	−2.265689
H	−0.948172	−0.013767	−0.517581
H	−0.942072	−0.004072	1.912869
H	1.230173	−0.001396	3.168326
H	3.348991	0.017915	1.954523
N	4.921550	−0.137908	−0.026662
H	5.862176	−0.263023	−2.394600
H	5.849663	−0.185556	−4.834625
H	3.691172	0.022710	−6.087934
H	1.539243	0.208492	−4.819093
O	5.161020	0.743027	0.812094
O	5.705221	−1.065007	−0.272335

Carbanion 1g
E Gibbs = −1876.0328 a.u.
E scf = −1875.9800 a.u.
Enthalpy = −1875.9562 a.u.

C	0.000046	−0.029766	−0.021078
C	0.002606	−0.062767	1.366717
C	1.236231	−0.106849	2.033869
C	2.430049	−0.057441	1.328705
C	2.457684	0.023999	−0.088115
C	1.198086	−0.038797	−0.759417
C	1.188858	−0.068098	−2.232078
C	2.214772	0.793527	−2.853072
C	3.490093	0.864866	−2.204928

C	3.650278	0.227460	−0.900857
C	4.504592	1.600574	−2.871302
C	4.261822	2.275557	−4.058369
C	2.989199	2.257063	−4.646607
C	1.984716	1.510328	−4.043788
C	0.339974	−0.845069	−2.966823
C	−0.547461	−1.948825	−2.468999
O	0.212885	−0.674519	−4.340689
F	−1.860801	−1.588586	−2.271197
F	−0.141633	−2.561377	−1.345294
F	−0.614627	−2.941267	−3.434234
H	0.191822	−1.543585	−4.765076
H	−0.943250	0.007357	−0.556653
H	−0.932438	−0.054426	1.919943
H	1.267683	−0.149843	3.120180
H	3.360657	−0.068344	1.877791
H	5.494081	1.647972	−2.440303
H	5.070218	2.838657	−4.519413
H	2.789511	2.806840	−5.562561
H	1.001453	1.470335	−4.493934
O	6.213655	−0.286513	−1.348046
O	5.147470	−1.121265	0.815543
C	5.913920	1.388554	0.658092
F	6.065981	2.457544	−0.156746
F	5.105706	1.772229	1.672638
F	7.124400	1.107808	1.187551
S	5.203968	−0.133148	−0.280852

Alkoxide 1g
E Gibbs = −1876.0129 a.u.
E scf = −1875.9589 a.u.
Enthalpy = −1875.9352 a.u.

C	0.021600	0.070333	−0.015419
C	1.387667	0.200696	0.002631
C	1.957237	1.493362	0.054457
C	1.161774	2.612844	0.113130
C	−0.267278	2.526982	0.121873
C	−0.855514	1.202526	0.028881
C	−2.268199	1.033271	0.010802
C	−3.085086	2.124093	0.417568
C	−2.543808	3.464823	0.502993
C	−1.148761	3.649324	0.226158
C	−3.448133	4.510521	0.871282
C	−4.767707	4.253789	1.161551
C	−5.283898	2.937396	1.112531
C	−4.459376	1.901966	0.754750
C	−2.899163	−0.343838	−0.357987
C	−3.619757	−0.217630	−1.749078
O	−3.655664	−0.851334	0.612814
F	−3.947852	−1.452120	−2.221186

F	−2.811397	0.345892	−2.709935
F	−4.769728	0.503788	−1.758885
H	−2.060027	−1.001516	−0.681010
H	−0.411613	−0.920169	−0.005440
H	2.021452	−0.681522	−0.000464
H	3.037680	1.612021	0.073279
H	1.637934	3.578500	0.167496
H	−3.095172	5.527068	0.937430
H	−5.409708	5.079466	1.459572
H	−6.317839	2.743441	1.384584
H	−4.778981	0.863685	0.770952
O	−1.565647	6.242793	−0.424266
O	0.749751	5.293712	−0.785981
C	0.014315	5.916919	1.691410
F	−1.023602	5.934983	2.545643
F	0.976880	5.137264	2.217263
F	0.495976	7.167741	1.584812
S	−0.513722	5.293677	−0.033367

Carbanion 2b
E Gibbs = −747.9674 a.u.
E scf = −747.9268 a.u.
Enthalpy = −747.9110 a.u.

C	0.000000	0.000000	0.000000
C	0.000000	0.000000	1.401248
C	1.235858	0.000000	2.070252
C	2.428905	0.055419	1.359903
C	2.452432	0.101915	−0.065025
C	1.185738	−0.005801	−0.745306
C	1.222218	−0.131920	−2.220290
C	2.304364	0.648002	−2.870859
C	3.566134	0.729799	−2.161771
C	3.652564	0.306569	−0.811339
C	4.661915	1.310462	−2.877983
C	4.507694	1.864346	−4.144480
C	3.253934	1.885279	−4.773244
C	2.171082	1.272404	−4.117753
C	0.464316	−1.030932	−2.914043
C	−0.525899	−2.013657	−2.366619
O	0.581868	−1.207403	−4.287462
H	−1.566888	−1.698731	−2.534577
H	−0.388405	−2.162102	−1.294398
H	−0.394556	−2.970680	−2.887530
H	1.344364	−0.683103	−4.587668
H	−0.948976	0.004975	−0.530923
H	−0.936816	−0.005289	1.953097
H	1.263481	−0.023040	3.159193
H	3.381683	0.085775	1.878136
N	4.899392	0.389540	−0.080635
H	5.639726	1.343056	−2.404931

H	5.371482	2.305528	−4.640448
H	3.118628	2.351842	−5.745321
H	1.186424	1.315768	−4.581187
H	5.266560	1.343577	−0.057162
H	5.618837	−0.184878	−0.514066

Alkoxide 2b
E Gibbs = −747.9613 a.u.
E scf = −747.9202 a.u.
Enthalpy = −747.9045 a.u.

C	0.000000	0.000000	0.000000
C	0.000000	0.000000	1.374206
C	1.228531	0.000000	2.087412
C	2.413881	0.018724	1.394118
C	2.455497	0.036249	−0.038526
C	1.205651	0.007690	−0.780291
C	1.182144	−0.008545	−2.203685
C	2.435354	−0.018660	−2.872427
C	3.689815	−0.007067	−2.141648
C	3.682914	0.066470	−0.733447
C	4.916891	−0.079222	−2.881616
C	4.924565	−0.129788	−4.254636
C	3.700073	−0.098232	−4.975528
C	2.499073	−0.042097	−4.309169
C	−0.185213	−0.062367	−2.995122
C	−0.661676	−1.562004	−2.950525
O	−0.195677	0.423901	−4.248747
H	−1.645347	−1.607985	−3.432299
H	−0.734170	−1.984332	−1.936242
H	0.031083	−2.176679	−3.538595
H	−0.903601	0.491093	−2.339404
H	−0.947706	−0.001049	−0.521774
H	−0.942810	−0.004277	1.916119
H	1.231625	−0.024440	3.174782
H	3.341636	−0.021139	1.957790
N	4.907195	0.112909	−0.014441
H	5.865485	−0.138829	−2.355209
H	5.871629	−0.196919	−4.787419
H	3.715889	−0.113629	−6.063426
H	1.523884	0.021543	−4.802023
H	4.825620	0.615081	0.861084
H	5.655541	0.528105	−0.555341

Carbanion 2c
E Gibbs = −807.1254 a.u.
E scf = −807.0823 a.u.
Enthalpy = −807.0650 a.u.

C	−1.420496	−2.249019	−0.452062
C	−2.818448	−2.283263	−0.566835

C	−3.539032	−1.095025	−0.361183
C	−2.880374	0.099698	−0.095200
C	−1.458711	0.161104	−0.006170
C	−0.728095	−1.077778	−0.127229
C	0.733132	−1.022221	0.115606
C	1.385973	0.227724	−0.352498
C	0.628178	1.451646	−0.209570
C	−0.741464	1.377890	0.129512
C	1.302867	2.678104	−0.494232
C	2.607978	2.697118	−0.968793
C	3.309069	1.501758	−1.202529
C	2.678810	0.285128	−0.892482
C	1.398393	−1.939705	0.876997
C	0.833032	−3.143309	1.567679
O	2.748953	−1.821055	1.179462
H	3.046673	−0.951097	0.861664
H	−0.851911	−3.159980	−0.622619
H	−3.328630	−3.212208	−0.808658
H	−4.625801	−1.100102	−0.435301
H	−3.442014	1.023026	0.009237
H	0.751811	3.605592	−0.372295
H	3.081283	3.653673	−1.186734
H	4.315731	1.511750	−1.611799
H	3.199258	−0.645781	−1.113446
H	−0.251654	−3.072834	1.664695
H	1.065585	−4.079984	1.038844
H	1.284716	−3.219978	2.564881
O	−1.473336	2.580290	0.219055
C	−1.649540	3.024128	1.555556
H	−2.224836	3.957985	1.514957
H	−2.202533	2.286177	2.155491
H	−0.683779	3.216390	2.046281

Alkoxide 2c
E Gibbs = −807.1166 a.u.
E scf = −807.0736 a.u.
Enthalpy = −807.0566 a.u.

C	−1.580569	−2.263376	−0.106954
C	−2.955442	−2.247357	−0.105172
C	−3.660696	−1.012759	−0.143415
C	−2.962990	0.168405	−0.180809
C	−1.531145	0.185804	−0.171512
C	−0.792924	−1.063417	−0.133617
C	0.634693	−1.079225	−0.114843
C	1.309830	0.169997	−0.207166
C	0.576109	1.422439	−0.241569
C	−0.823985	1.396647	−0.190725
C	1.294681	2.659598	−0.315271
C	2.667422	2.673700	−0.342883
C	3.397666	1.452614	−0.311000

C	2.745195	0.243044	−0.258776
C	1.420276	−2.433436	0.069142
C	1.332553	−2.769954	1.605293
O	2.683824	−2.493274	−0.380883
H	0.774924	−3.198255	−0.430950
H	−1.068297	−3.216877	−0.100687
H	−3.506202	−3.184921	−0.091954
H	−4.748362	−1.006989	−0.159839
H	−3.484747	1.118208	−0.239827
H	0.727449	3.583042	−0.372588
H	3.197689	3.622375	−0.408860
H	4.484614	1.477602	−0.354401
H	3.245650	−0.729174	−0.276517
H	0.306590	−2.806305	2.002371
H	1.813144	−3.743930	1.752052
H	1.904662	−2.022701	2.169080
O	−1.534543	2.600261	−0.189076
C	−1.771377	3.124244	1.116040
H	−2.310274	4.067700	0.984221
H	−2.382740	2.436661	1.716306
H	−0.828715	3.314054	1.645896

Carbanion 2d
E Gibbs = −731.9195 a.u.
E scf = −731.8787 a.u.
Enthalpy = −731.8627 a.u.

C	2.060598	1.480154	−0.515788
C	3.406759	1.094642	−0.597512
C	3.741876	−0.224221	−0.256389
C	2.757482	−1.138329	0.105457
C	1.373629	−0.784317	0.166600
C	1.057034	0.600998	−0.096948
C	−0.347727	1.014696	0.111939
C	−1.340432	−0.016825	−0.273971
C	−0.990211	−1.393445	0.010468
C	0.332828	−1.732754	0.407241
C	−2.024535	−2.360994	−0.203653
C	−3.265376	−2.020549	−0.729937
C	−3.554749	−0.697125	−1.096688
C	−2.576886	0.283256	−0.862279
C	−0.705568	2.154687	0.771150
C	0.195610	3.188620	1.375124
O	−2.029851	2.476723	1.043368
H	−2.576652	1.713808	0.787912
H	1.781781	2.495980	−0.785726
H	4.167127	1.803017	−0.916564
H	4.780428	−0.550278	−0.299802
H	3.050475	−2.163116	0.312794
H	−1.822101	−3.405295	0.014016
H	−4.008785	−2.801731	−0.884227

H	−4.506762	−0.432417	−1.549032
H	−2.772497	1.306517	−1.179916
H	1.211343	2.808977	1.497061
H	0.246721	4.105924	0.769334
H	−0.206624	3.476062	2.355014
C	0.675195	−3.156031	0.772727
H	0.905863	−3.803351	−0.096254
H	1.548760	−3.199861	1.434962
H	−0.147471	−3.640118	1.313291

Alkoxide 2d
E Gibbs = −731.9111 a.u.
E scf = −731.8678 a.u.
Enthalpy = −731.8518 a.u.

C	2.239575	1.453398	−0.083091
C	3.547004	1.039942	0.006423
C	3.846662	−0.344744	0.094867
C	2.827261	−1.264704	0.078153
C	1.447336	−0.877542	−0.014721
C	1.133450	0.538782	−0.085079
C	−0.216770	0.993606	−0.147456
C	−1.239802	0.008753	−0.126594
C	−0.927131	−1.409822	−0.035565
C	0.416817	−1.845335	−0.011663
C	−2.028743	−2.329782	0.054277
C	−3.333820	−1.903004	0.035596
C	−3.635899	−0.520563	−0.092962
C	−2.622609	0.402466	−0.179798
C	−0.547484	2.538466	−0.176687
C	−0.422873	3.034531	1.313379
O	−1.710188	2.920383	−0.731513
H	0.323138	2.994868	−0.709249
H	2.031878	2.512386	−0.158624
H	4.350231	1.772956	0.003921
H	4.879719	−0.677275	0.171506
H	3.076012	−2.317576	0.152247
H	−1.832786	−3.390997	0.158117
H	−4.138622	−2.632649	0.112186
H	−4.673461	−0.195451	−0.135146
H	−2.779611	1.476392	−0.320413
H	0.543156	2.806640	1.788475
H	−0.576624	4.119728	1.308052
H	−1.224855	2.582815	1.909753
C	0.772697	−3.318044	0.040422
H	1.603023	−3.551943	−0.634618
H	1.077142	−3.639104	1.047739
H	−0.059271	−3.953765	−0.264311

Carbanion 2e
E Gibbs = −784.9108 a.u.
E scf = −784.8691 a.u.
Enthalpy = −784.8530 a.u.

C	−0.261727	0.235077	−1.187455
C	1.134153	0.165388	−1.239320
C	1.883224	1.165229	−0.601566
C	1.247448	2.233810	0.021401
C	−0.166583	2.341641	0.053782
C	−0.927589	1.267423	−0.511021
C	−2.398241	1.307492	−0.329402
C	−2.985860	2.670850	−0.383463
C	−2.223369	3.737103	0.199667
C	−0.852692	3.502489	0.574656
C	−2.854704	5.003676	0.318806
C	−4.130398	5.235971	−0.182533
C	−4.830561	4.220195	−0.849936
C	−4.244782	2.951638	−0.942506
C	−3.132217	0.223526	0.051628
C	−2.643447	−1.167929	0.322981
O	−4.489829	0.294757	0.322057
H	−4.754754	1.229765	0.298205
H	−0.852807	−0.530165	−1.683681
H	1.624742	−0.651373	−1.762636
H	2.970699	1.124078	−0.613796
H	1.838552	3.024361	0.475636
C	−0.120302	4.521147	1.214330
H	−2.304821	5.813115	0.791138
H	−4.572337	6.224965	−0.078420
H	−5.807719	4.407554	−1.287040
H	−4.764191	2.169166	−1.492881
N	0.487524	5.368886	1.757627
H	−2.888111	−1.859976	−0.495783
H	−1.563723	−1.191439	0.475338
H	−3.145545	−1.545294	1.221981

Alkoxide 2e
E Gibbs = −784.8859 a.u.
E scf = −784.8438 a.u.
Enthalpy = −784.8278 a.u.

C	−2.202254	−1.656948	−0.249592
C	−3.518570	−1.260396	−0.210175
C	−3.844777	0.114881	−0.090330
C	−2.842104	1.051291	−0.022370
C	−1.465420	0.670672	−0.058091
C	−1.114783	−0.726367	−0.163865
C	0.255638	−1.148318	−0.173092
C	1.267767	−0.145405	−0.138836
C	0.929043	1.257070	−0.026422

C	−0.434439	1.641899	0.022541
C	1.977982	2.222270	0.051283
C	3.297187	1.835644	0.018574
C	3.640781	0.464640	−0.108363
C	2.660133	−0.496380	−0.196424
C	0.613484	−2.676333	−0.160967
C	0.393240	−3.142193	1.330195
O	1.811105	−3.055798	−0.625750
H	−0.212820	−3.156223	−0.739599
H	−1.977868	−2.709250	−0.361660
H	−4.309575	−2.002251	−0.284447
H	−4.885278	0.429319	−0.063972
H	−3.085859	2.106331	0.060153
C	−0.775652	3.019199	0.158167
H	1.715990	3.272642	0.137198
H	4.079508	2.589941	0.076447
H	4.687785	0.174467	−0.157604
H	2.854554	−1.562882	−0.334639
N	−1.058329	4.148393	0.266704
H	−0.604570	−2.918022	1.734036
H	0.561209	−4.224258	1.352498
H	1.150006	−2.669163	1.966977

Carbanion 2f
E Gibbs = −897.1772 a.u.
E scf = −897.1345 a.u.
Enthalpy = −897.1178 a.u.

C	0.000000	0.000000	0.000000
C	0.000000	0.000000	1.395169
C	1.228770	0.000000	2.064509
C	2.428852	0.025647	1.357183
C	2.456093	0.047470	−0.059686
C	1.196647	−0.012439	−0.732155
C	1.218751	−0.063669	−2.209704
C	2.242627	0.824488	−2.808201
C	3.521228	0.877608	−2.169073
C	3.664872	0.209160	−0.875435
C	4.523675	1.649314	−2.810015
C	4.263066	2.368260	−3.974950
C	2.990906	2.362060	−4.555919
C	1.990780	1.589604	−3.960503
C	0.502972	−0.950369	−2.950920
C	−0.438674	−2.006699	−2.455670
O	0.617336	−1.039853	−4.326621
H	−1.484377	−1.754985	−2.681108
H	−0.344598	−2.156860	−1.380081
H	−0.216275	−2.947518	−2.973320
H	1.332188	−0.447315	−4.613428
H	−0.942261	0.017843	−0.541065
H	−0.937606	0.003132	1.945886

H	1.257389	−0.005290	3.152237
H	3.369362	0.027960	1.888579
N	4.912751	−0.140209	−0.385374
H	5.510213	1.671672	−2.370615
H	5.063548	2.953796	−4.422379
H	2.777794	2.946483	−5.447501
H	0.983469	1.606799	−4.371814
O	5.024308	−0.685580	0.756050
O	5.954393	0.047093	−1.088977

Alkoxide 2f
E Gibbs = −897.1372 a.u.
E scf = −897.0937 a.u.
Enthalpy = −897.0769 a.u.

C	0.000000	0.000000	0.000000
C	0.000000	0.000000	1.373570
C	1.229954	0.000000	2.075548
C	2.418690	0.021939	1.388423
C	2.457174	0.026578	−0.043485
C	1.207408	−0.007461	−0.773385
C	1.178928	−0.062181	−2.205413
C	2.419004	−0.038507	−2.903809
C	3.687437	−0.063232	−2.198492
C	3.665895	−0.027672	−0.782543
C	4.892294	−0.065850	−2.968326
C	4.857278	−0.049039	−4.343055
C	3.623215	−0.024309	−5.038046
C	2.439835	−0.005043	−4.339472
C	−0.188578	−0.207164	−2.963200
C	−0.582820	−1.727776	−2.821968
O	−0.269893	0.206989	−4.235114
H	−1.573719	−1.840937	−3.274345
H	−0.607901	−2.097587	−1.787151
H	0.124259	−2.334104	−3.399773
H	−0.916100	0.344865	−2.322876
H	−0.947844	0.011975	−0.519761
H	−0.941173	0.010128	1.917183
H	1.239020	−0.002666	3.162792
H	3.348729	0.049861	1.939496
N	4.927159	−0.047676	−0.063067
H	5.846704	−0.095660	−2.460093
H	5.794358	−0.043989	−4.896348
H	3.612987	0.004387	−6.125095
H	1.451539	0.060126	−4.803455
O	5.108434	0.772887	0.854816
O	5.792682	−0.878086	−0.389353

Carbanion 2g
E Gibbs = −1578.2756 a.u.
E scf = −1578.2259 a.u.
Enthalpy = −1578.2044 a.u.

C	2.109274	2.419464	−1.170076
C	1.408375	3.495156	−1.715578
C	0.015561	3.407105	−1.819497
C	−0.655658	2.252355	−1.423764
C	0.033734	1.147741	−0.870642
C	1.445413	1.273387	−0.705084
C	2.156098	0.146575	−0.059612
C	1.661912	−1.185860	−0.485619
C	0.249706	−1.347118	−0.627131
C	−0.590941	−0.146019	−0.547918
C	−0.224305	−2.645964	−0.930820
C	0.645860	−3.717448	−1.119213
C	2.031322	−3.534801	−1.055019
C	2.525256	−2.265045	−0.748099
C	3.058653	0.296451	0.945639
C	3.521392	1.575189	1.576900
O	3.640238	−0.776605	1.595155
H	3.221011	−1.593479	1.277517
H	3.192880	2.460317	−1.098536
H	1.939039	4.378996	−2.060875
H	−0.553972	4.232575	−2.240989
H	−1.728985	2.188976	−1.543113
H	−1.290967	−2.799906	−1.024437
H	0.235034	−4.697456	−1.351695
H	2.714007	−4.357974	−1.249561
H	3.600060	−2.095505	−0.737317
O	−2.756746	−1.566756	0.047575
O	−2.981039	0.963510	−0.197392
C	−1.973109	−0.019896	2.019157
S	−2.148094	−0.218518	0.119834
F	−1.203900	−1.000069	2.545415
F	−1.396088	1.160654	2.338084
F	−3.172415	−0.066610	2.645010
H	3.494774	1.458530	2.666776
H	2.890857	2.416969	1.290940
H	4.559617	1.808547	1.302930

Alkoxide 2g
E Gibbs = −1578.2340 a.u.
E scf = −1578.1844 a.u.
Enthalpy = −1578.1628 a.u.

C	1.964886	2.792546	0.516201
C	1.146827	3.888494	0.672530
C	−0.235421	3.753188	0.419780
C	−0.774472	2.539184	0.051444

C	0.031505	1.368926	−0.094179
C	1.451308	1.521706	0.121814
C	2.339808	0.412300	−0.046252
C	1.772441	−0.903206	0.003942
C	0.365116	−1.100387	−0.241091
C	−0.467894	0.062485	−0.434858
C	−0.107008	−2.448147	−0.252226
C	0.727528	−3.513859	0.010239
C	2.090105	−3.308361	0.316624
C	2.592420	−2.027207	0.312760
C	3.837759	0.653060	−0.166280
C	4.552744	−0.107861	−1.310632
O	4.247512	0.375745	1.104105
H	3.972318	1.721205	−0.457479
H	3.015339	2.858636	0.769904
H	1.556654	4.836876	1.008880
H	−0.896924	4.607876	0.539985
H	−1.838648	2.476931	−0.114945
H	−1.148479	−2.646337	−0.452631
H	0.312707	−4.519134	0.013927
H	2.727429	−4.146632	0.585488
H	3.605505	−1.813750	0.632393
O	−2.228192	−1.363345	−1.879546
O	−2.560668	1.145484	−1.726543
C	−3.281617	−0.355804	0.331292
S	−2.070555	−0.107416	−1.126568
F	−2.971756	−1.452997	1.048732
F	−3.269576	0.701718	1.166601
F	−4.536790	−0.503775	−0.134793
H	5.615079	0.160487	−1.267036
H	4.159119	0.191780	−2.293048
H	4.474652	−1.194703	−1.226363

Figure S1. Electrophilic Fukui Function for the alkoxides and carbanions of 1c, 1d, 1e, 1g, 2c, 2d, 2e and 2g.

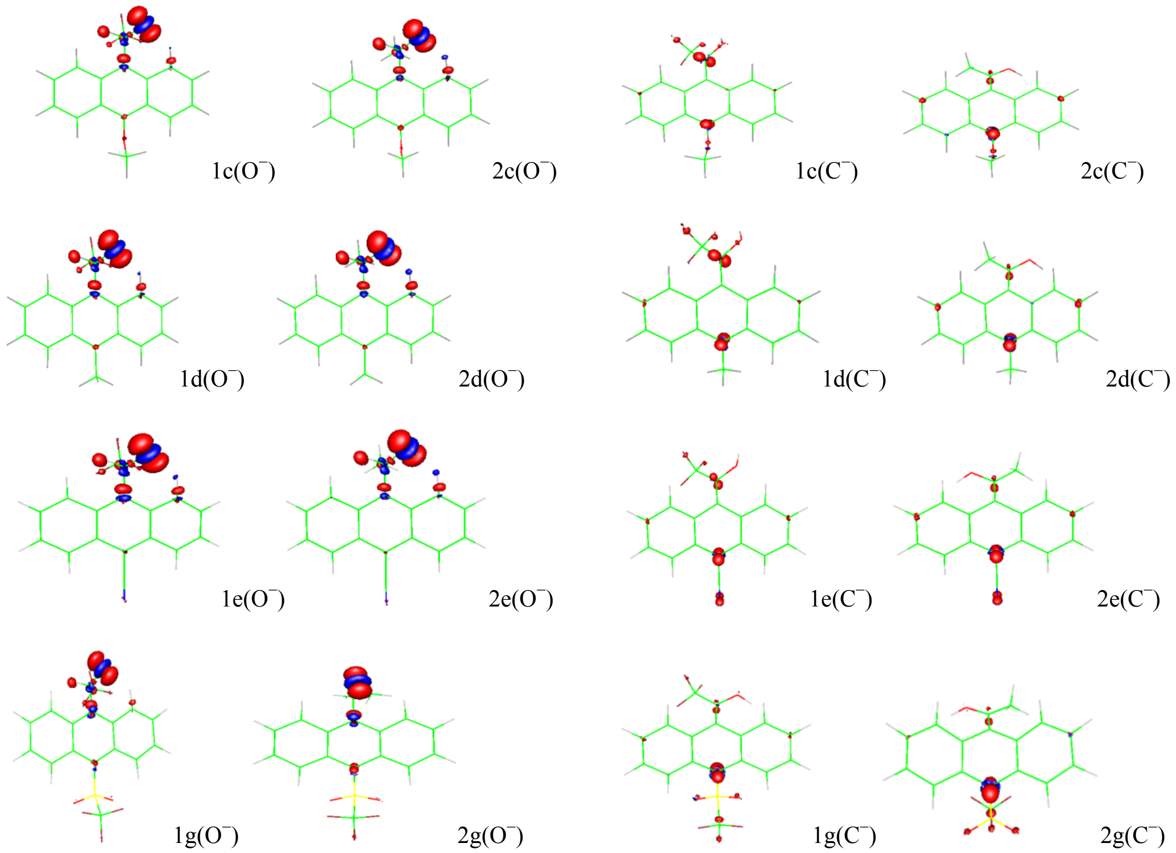

Figure S2. MEP for the alkoxides and carbanions of 1c, 1d, 1e, 1g, 2c, 2d, 2e and 2g.

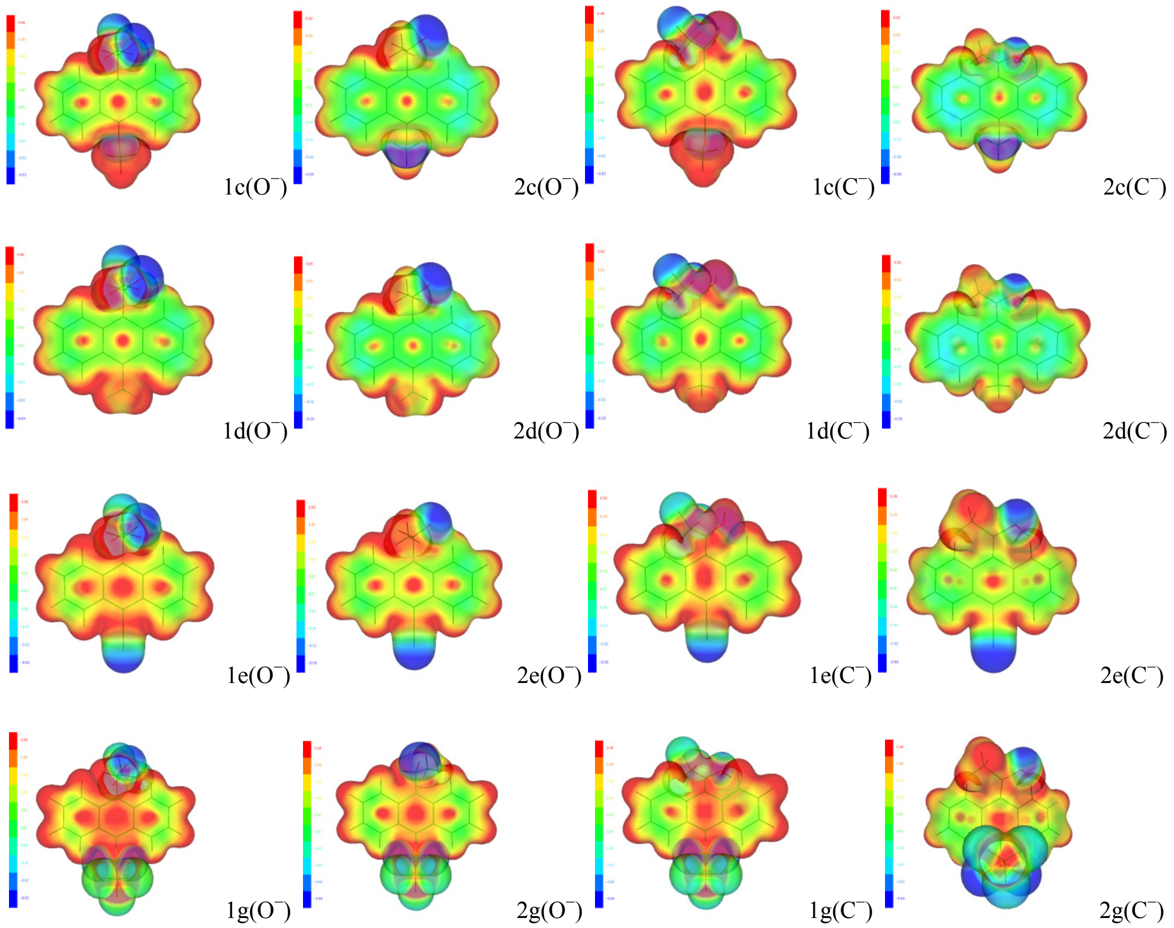

Supplement: Supplementary file 1 [file molecules-18-10254-s001.pdf]
